# Time series analysis of COVID-19's impact on physician and dentist visits in Iran

**DOI:** 10.1038/s41598-024-67238-9

**Published:** 2024-07-16

**Authors:** Satar Rezaei, Hesam Ghiasvand, Heather Brown

**Affiliations:** 1 Research Center for Environmental Determinants of Health, Health Institute, Kermanshah University of Medical Sciences, Kermanshah, Iran; 2 Research Centre for Healthcare & Communities, Research Institute for Health & Wellbeing, Coventry University, Coventry, UK; 3 Division of Health Research, Lancaster University, Lancaster, UK

**Keywords:** Interrupted time series analysis, COVID-19, Physician, Dentist, Visit, Iran, Health care, Health care economics

## Abstract

This study aimed to assess the impact of the COVID-19 pandemic on general practitioner (GP), specialist, and dentist visits among 40 million Iranians covered by the Social Security Organization (SSO). A monthly interrupted time series analysis was conducted over a period of 72 months, including—47 months before the pandemic and 25 months after its onset. The outcomes variables were monthly number of GP, specialist, and dentist visits per 1000 SSO-insured individuals. The analysis was performed by total visits, visits to the SSO direct sector, and visits to the indirect sectors. The study found that in the first month of the pandemic, the number of visits per 1000 insured individuals significantly decreased for visits to GPs (by 51.12, 95% CI −64.42 to −37.88), visits to specialists (by 39.11, 95% CI −51.61 to −26.62), and visits to dentists (by 6.67, 95% CI −8.55 to −4.78). However, during the subsequent months of the pandemic, there was a significant increase in the number of monthly visits for all three categories, with GPs experiencing the highest increase (1.78 visits per 1000 insured), followed by specialists (1.32 visits per 1000 insured), and dentists (0.05 visits per 1000 insured). Furthermore, prior to the pandemic, the number of monthly GP visits per 1000 insured individuals was statistically significantly lower in the indirect sector compared to the direct sector (45.79, 95% CI −52.69 to −38.89). Conversely, the direct sector exhibited lower rates of specialist visits (25.84 visits per 1000 insured individuals, 95% CI 22.87 to 28.82) and dentist visits (0.75 visits per 1000 insured individuals, 95% CI 0.12 to 1.36) compared to the indirect sector. Additionally, the study found that in the first month of the pandemic, the monthly number of GP visits in the indirect sector significantly increased by 34.44 times (95% CI 24.81 to 44.08) compared to the direct sector. For specialist visits and dentist visits, the increase was 3.41 (95% CI −5.87 to 12.69) and 5.01 (95% CI 3.48 to 6.53) per 1000 insured individuals, respectively. Overall, the findings of this study demonstrate statistically significant disruptions in GP, specialist, and dentist visits during the COVID-19 pandemic, although some recovery was observed. Both the direct and indirect sectors experienced decreased visits.

## Introduction

The COVID-19 pandemic effected the behavior of patients, providers, and policymakers in the health system. This included the, altering of service delivery patterns because of lockdowns and stay-at-home orders. Because of these restrictions care needed to be offered in different ways such as via telemedicine. Research has demonstrated changes in hospital admission rate, bed occupancy rate, average hospital stay duration, emergency department visits, and physician and dentist visits^1–7^. In addition, demand for providing services remotely and through telemedicine has increased after the pandemic^8,9^. A study found that during the pandemic video visits increased from 0.1% of patient interactions to 43.5% and number of weekly telehealth visits increased on average from 120 to 8000^9^. However, the extent of the impact has varied among different health systems, and it is crucial to understand how context related to health system design may have impacted on these variations for future pandemic preparedness.

The COVID-19 pandemic meant that resources had to be reallocated to treat infected patients with the virus and reduce the spread. This resulted in significant disruptions to regular care and services for those with chronic diseases and conditions unrelated to coronavirus. Many healthcare workers were reassigned from their normal duties to assist with the COVID-19 response^10,11^. Furthermore, the pandemic dramatically impacted healthcare utilization by non-COVID patients. Many individuals with chronic illnesses such as hypertension and history of stroke were forced to cancel or postpone routine appointments and follow-up visits^12,13^. Some patients missed scheduled check-ups entirely during the peak of the outbreak for fear of catching the virus. The overwhelming demand of the public health emergency led to the deferring of regular preventive care and maintenance of non-critical conditions which were considered non-essential^14^. However, while the impact of COVID-19 on physician and dentist visits has been extensively studied^7,15,16^, its specific effects on these types of health services have not yet been examined in Iran either at the national or subnational levels. This analysis identifies opportunities for more effective and efficient support of the healthcare workforce, systems, and patient care delivery for future public health crises across a lower and middle income country.

In Iran, healthcare is provided by three main sectors: public, private, and non-profit/charity. The public sector plays a significant role in primary, secondary, and tertiary care, while the private sector focuses on secondary and tertiary care in urban areas. Non-government organizations and charities also offer healthcare services for patients with chronic or severe illnesses. The Ministry of Health and Medical Education governs the national health system, and medical universities affiliated with the ministry oversee healthcare at the regional level. Healthcare funding in Iran comes from government budgets, public and private health insurance, and out-of-pocket payments. The Iranian Health Insurance Organization, the Social Security Organization, the Armed Forces Medical Service Organization, and the Imam Khomeini Relief Foundation are among the public insurers. Additionally, some organizations such as oil companies and banks have their own insurance schemes, and private health insurance options are also available. The Social Security Organization (SSO) is the largest social insurance entity in the country that covers more than half of the population (exactly 44,150,091 in 2021) and this makes it an appropriate candidate of analysis in terms of realizing the impacts of the COVID-19 pandemic on utilizing of the organization’s obligations for routine health care^17–22^.

The aim of the study is to investigate the impact of the COVID-19 pandemic on physician (general practitioner and specialist) and dentist visits among Social Security Organization (SSO) insured individuals in Iran at the national and provincial levels and in both the direct and indirect sectors (see supplementary appendix 1 for a description of the country’s health system and the SSO). Focusing on GPs, specialists, and dentists is crucial due to their critical roles in providing primary, preventative and specialized care. Understanding the pandemic's impact on their practices helps identify challenges and inform strategies to mitigate disruptions in healthcare delivery in future pandemic scenarios. Additionally, investigating the impact on these health services provides insights into the resilience and capacity of the broader healthcare system, enabling the identification of vulnerabilities and the design of interventions for future public health emergencies. Although we anticipate that the results of our study will provide a picture of the extent to which COVID-19 has interrupted routine health care by GPs, specialists and dentist in Iran, we believe the findings provide important insights that can inform preparation and planning for future pandemics in other countries as well. The disruptions to healthcare access and delivery during COVID-19 highlighted the need for robust pandemic preparedness plans that can help maintain essential health services during public health crises. While the specific impacts may vary given differences in healthcare systems, resources, and population demographics across countries, this study sheds light on some of the common challenges around healthcare utilization that may emerge during a pandemic. By understanding these potential pressure points, countries can use the findings from our work to strengthen their own pandemic preparedness efforts.

## Methods

This is a quasi-experimental study on people who are under the SSO coverage in Iran at the national and provincial levels. The SSO cover approximately 44,150,091 of people equals to more than 52% of the country population in 2021. Data on monthly GP, specialist, and dentist visits was extracted from March 2016 to March 2022 (47 months pre-pandemic and 25 months during the pandemic). The data came from the SSO's insurance information system and was categorized into direct, indirect, and total sectors. The first confirmed case of COVID-19 in Iran was reported on 19 February 2020, marking the onset of the outbreak in the country. Therefore, the pre-pandemic period was defined as 20 March 2016 to 19 Feb 2020 and the pandemic period was 19 February 2020 to 20 March 2021. The three outcome variables were GP visits, specialist visits, and dentist visits per 1000 insured.

To analysis the data, we started with descriptive statistics and conducted univariate analysis using the Mann–Whitney test. The purpose was to compare the average GP visits, specialist visits, and dentist visits per 1000 insured 47 months before the pandemic and 25 months after its onset. Next, we employed an interrupted time series analysis approach (ITSA) for analyzing the data. This approach is chosen because of its capabilities in demonstrating the impacts of an intervention particularly at population level where there would be no control group as it provides some internal validity^24–27^. Additionally, ITSA has been used to examine the effects of unplanned events such as the COVID-19 pandemic on population health trends^3,28–32^. A single and multiple group ITSA was utilized. Single group ITSA was applied to examine the impact of the pandemic on the outcome variables (GP, specialist and dentist visit) for the whole covered population by SSO regardless of the route of the care that they have received (direct or indirect) and multiple group ITSA was used to assess the impact of the pandemic on the outcome variables (GP, specialist and dentist visit) in indirect sector versus direct sector.

We used the following model for single group analysis^33^:

$${Y}_{t}={\beta }_{0}+{\beta }_{1}*{time}_{t}+{\beta }_{2}{*\text{COVID}19}_{t}+{\beta }_{3}*{timeduringCOVID19}_{t}+{\varepsilon }_{t}$$

where ${Y}_{t}$ shows visits either for GP, or specialist, or dentist in month t. ${time}_{t}$ is a time trend variable from 1 to the total number of months. ${\text{COVID}-19}_{t}$ is a binary indicator (proxy) variable coded 0 for pre-pandemic and 1 for pandemic period (coded 1). ${timeduringCOVID19}_{t}$ indicates the number of months after the start of the pandemic. ${\beta }_{0}$ estimates the baseline level health visit (for the three services) at time zero. ${\beta }_{1}$ estimates the pre-pandemic trend. ${\beta }_{2}$ estimates the level change after the start of COVID-19. ${\beta }_{3}$ estimates the change in trend during the pandemic compared to the pre-pandemic trend.

We used the multiple groups ITSA to investigate the effects of the COVID-19 pandemic on visit of GP, specialist and dentist in the direct sector versus indirect sector. The following segmented regression model was used for the analysis^34^:

$${\text{Y}}_{t}={\upbeta }_{0}+{\upbeta }_{1}{T}_{t}+{\upbeta }_{2}{\text{X}}_{t}+{\upbeta }_{3}{T}_{t}{\text{X}}_{t}+{\upbeta }_{4}\text{Z}+{\upbeta }_{5}\text{Z}{T}_{t}+{\upbeta }_{6}{\text{ZX}}_{t}+{\upbeta }_{7}\text{Z}{T}_{t}{\text{X}}_{t}+{\text{e}}_{t}$$

where ${\text{Y}}_{t}$ is the Outcome variable (e.g. GP visit, specialist visit and dentist visit). ${T}_{t}$ shows the time since start of study (continuous). ${X}_{t}$ presents the binary variable representing pre/during pandemic periods (0 = pre, 1 = during the pandemic). $Z$ is the binary variable representing direct vs indirect sectors (0 = direct, 1 = indirect). ${T}_{t}{\text{X}}_{t}$, $\text{Z}{T}_{t}$, ${\text{ZX}}_{t}$ $\text{Z}{T}_{t}{\text{X}}_{t}$ are interaction terms. ${\beta }_{0}$ estimates intercept (starting level of Y in direct sector). ${\beta }_{1}$ estimates pre-pandemic slope (trend in Y over time in direct sector). ${\beta }_{2}$ estimates the level change in Y in direct sector in the first month of the pandemic. ${\beta }_{3}$ is the difference in slope changes between during the pandemic and pre-pandemic periods (change in trend in Y during the pandemic in direct sector). ${\beta }_{4}$ estimates the difference in baseline Y between direct and indirect sectors. ${\beta }_{5}$ estimates the difference in pre-pandemic trends between direct and indirect sectors. ${\beta }_{6}$ estimates the difference in level change during the pandemic between direct and indirect sectors. ${\beta }_{7}$ estimates the differences in slope changes from pre-pandemic to pandemic periods between direct and indirect sectors. ${e}_{t}$ shows the error term of model.

Newey-West standard errors were used in the regression models to correct for autocorrelation and heteroscedasticity. The *actest* command was employed to identify the optimal lag length to control for autocorrelation in each outcome variable. The stationarity of the outcome variables was tested by the augmented Dickey-Fuller test. Stationarity was confirmed based on p-values less than 0.05 for each outcome variable^34^. We also examined the presence of seasonality for each outcome variable using the command in Stata v.17 "twoway line yvar xvar", but no seasonal patterns were detected in the dataset. The F-test was utilized to evaluate the overall significance of the regression model. This statistical test assesses whether the model as a whole contributes a significant improvement in explaining the variability observed in the dependent variable, compared to a model with no predictors. We have added the F value to the tables for clarity and transparency. All data analyses were performed using Stata version 17 (StataCorp LLC, College Station, Texas 77845 USA). A p-value less than 0.05 was considered statistically significant.

### Ethical approval and consent to participate

The research protocol was reviewed and approved by the Research Deputy of Kermanshah University of Medical Sciences, with the approval number IR.KUMS.REC.1402.533. The study protocol was also approved by the Social Security Research Institute, which granted permission to access and utilize the data for this analysis.

## Results

Table 1 presents descriptive statistics for GP, specialist, and dentist visits per 1000 insured population. The largest change occurred in visits with specialists (a decrease of 31.4%) in direct sector, followed by 21.6% for GP visits in the same sector, meanwhile the dentist’s visits decreased about 30.4% in the indirect sector.

**Table 1:** Descriptive of the outcome variables included in the study.

Outcome variables	Sector	Before the pandemic (SD^a^)	After the pandemic (SD)	% change
Average number of GP^b^ visit per 1000 insured)	Direct sector	92.3 (8.84)	72.4 (13.3)	–21.6
	Indirect sector	39.9 (4.6)	36.6 (6.5)	–8.3
	Total	132.2 (12.9)	108.4 (19.2)	–18.0
Average number specialist visit per 1000 insured)	Direct sector	35.7 (4.3)	24.5 (5.9)	–31.4
	Indirect sector	56.5 (5)	44.5 (7.9)	–21.2
	Total	92.2 (9.9)	69.2 (13.7)	–24.9
Average number dentist visit per 1000 insured)	Direct sector	5.5 (2.3)	5.4 (1.6)	–1.8
	Indirect sector	2.3 (0.5)	1.6 (0.6)	–30.4
	Total	7.8 (2.1)	6.7 (1.9)	–14.1

^a^SD is the standard deviation; ^b^GP is the general physician.

**Figure 1:**
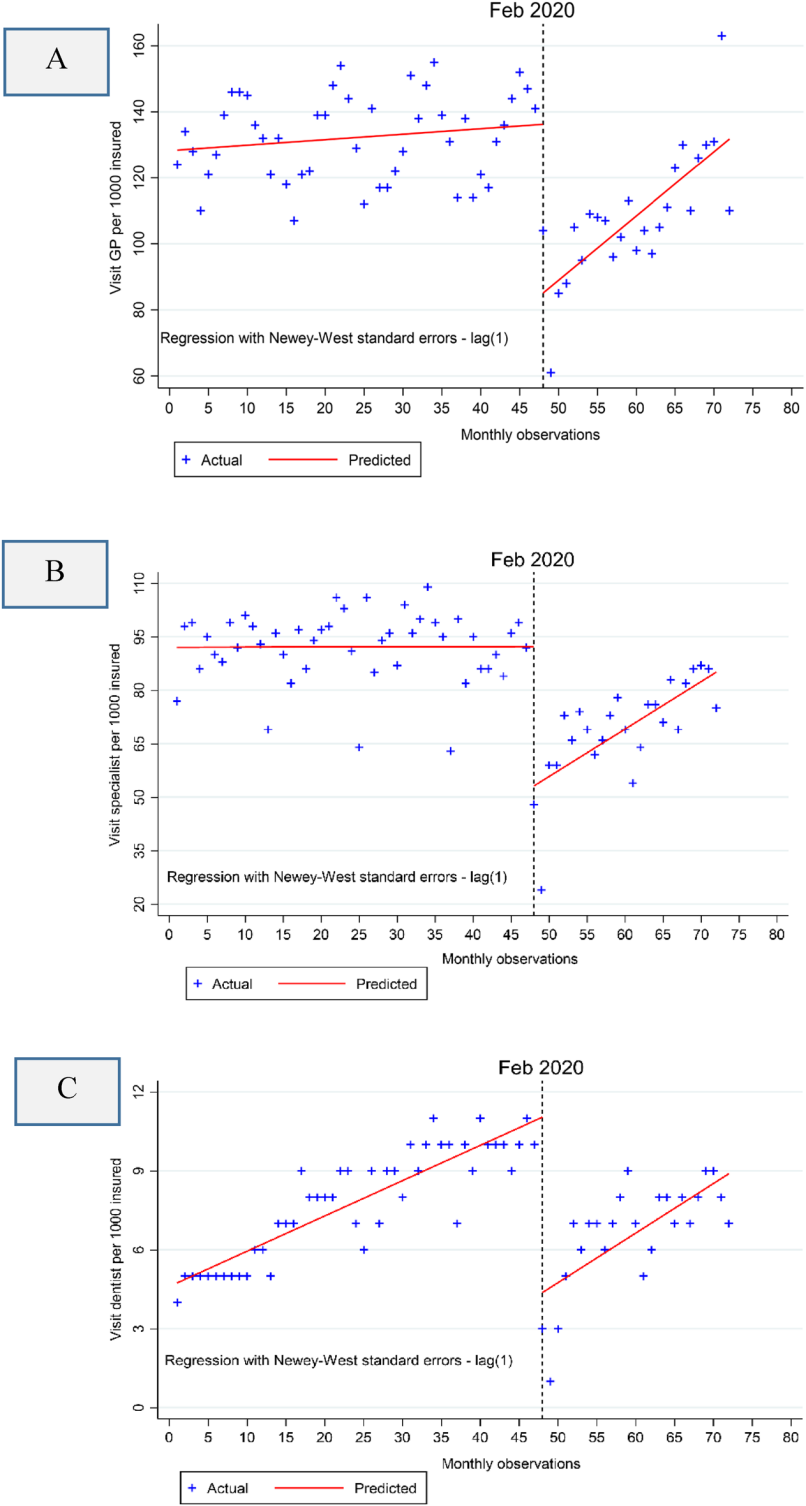
Single group ITSA with Newey–West standard errors for visits of GPs (**A**), specialists (**B**) and dentists (**C**). Note: The COVID-19 pandemic outbreak occurred in February 2020.

The results of the single group ITSA for both sectors are reported in Table 2. At the baseline, the mean of GP visits (128.43 [95% CI 120.22 to 136.64]) which exceeded the means for specialist (92.08[95% CI 87.63 to 96.53]) and dentist (4.74 [95% CI 4.25 to 5.23]) visits, and all were statistically significant (p-value < 0.001). Leading up to the pandemic, there was a slight increase in all types of visits, though it was marginal for specialist visits. As expected, in the first month of the pandemic, these visits experienced a significant and substantial decline, with the most significant drop observed in GP visits and specialist visits. All these decreases were statistically significant (p-value < 0.001). However, during the pandemic period, visits showed significant increases, with the highest increase for GP (1.78 [95% CI 0.94 to 2.64] per 1000 insured), followed by specialists (1.32 [95% CI 0.62 to 2.03] per 1000 insured), and dentists (0.05 [95% CI −0.06 to 0.17] per 1000 insured). These improvements were statistically significant for GP and specialist visits (p-value < 0.001), but not for dentists. The overall trend during the pandemic period indicates a stable increase in visits over time, following the same patterns for each type of care. Visual representations of these results can be found in Fig. 1A–C.

**Table 2:** Single group analysis of the effect of the COVID-19 pandemic on visits to GP, specialists, and dentists among those insured by the Social Security Organization in both direct and indirect sectors.

	GP^a^ visit		Specialist visit		Dentist visit	
	Coefficient (95% CI^b^)	p-value	Coefficient (95% CI)	p-value	Coefficient (95% CI)	p-value
Mean value at the baseline (β_0_)	128.43 (120.22 to 136.64)	< 0.001	92.08 (87.63 to 96.53)	< 0.001	4.74 (4.25 to 5.23)	< 0.001
Pre-trend (β_1_)	0.17 (−0.15 to 0.47)	0.292	0.004 (−0.16 to 0.17)	0.956	0.13 (0.11 to 0.15)	< 0.001
During-level change (β_2_)	−51.12 (−64.42 to −37.88)	< 0.001	−39.11 (−51.61 to −26.62)	< 0.001	−6.67 (−8.55 to −4.78)	< 0.001
During-trend change (β_3_)	1.78 (0.94 to 2.64)	< 0.001	1.32 (0.62 to 2.03)	< 0.001	0.05 (−0.06 to 0.17)	0.340
During-pandemic linear trend	1.94 (1.18 to 2.71)	< 0.001	1.33 (0.64 to 2.01)	0.0002	0.18 (0.08 to 0.29)	0.001
Model significance (F, P-value)	28.4 (< 0.001)		28.4 (< 0.001)		74.1 (< 0.001)	

^a^GP is the general physician; CI: confidence interval. β_0_ estimates the baseline level of the outcome at time zero; β_1_ estimates the pre-pandemic trend; β_2_ estimates the level change after the start of COVID-19 and β_3_ estimates the change in trend during the pandemic compared to the pre-pandemic trend.

**Figure 2:**
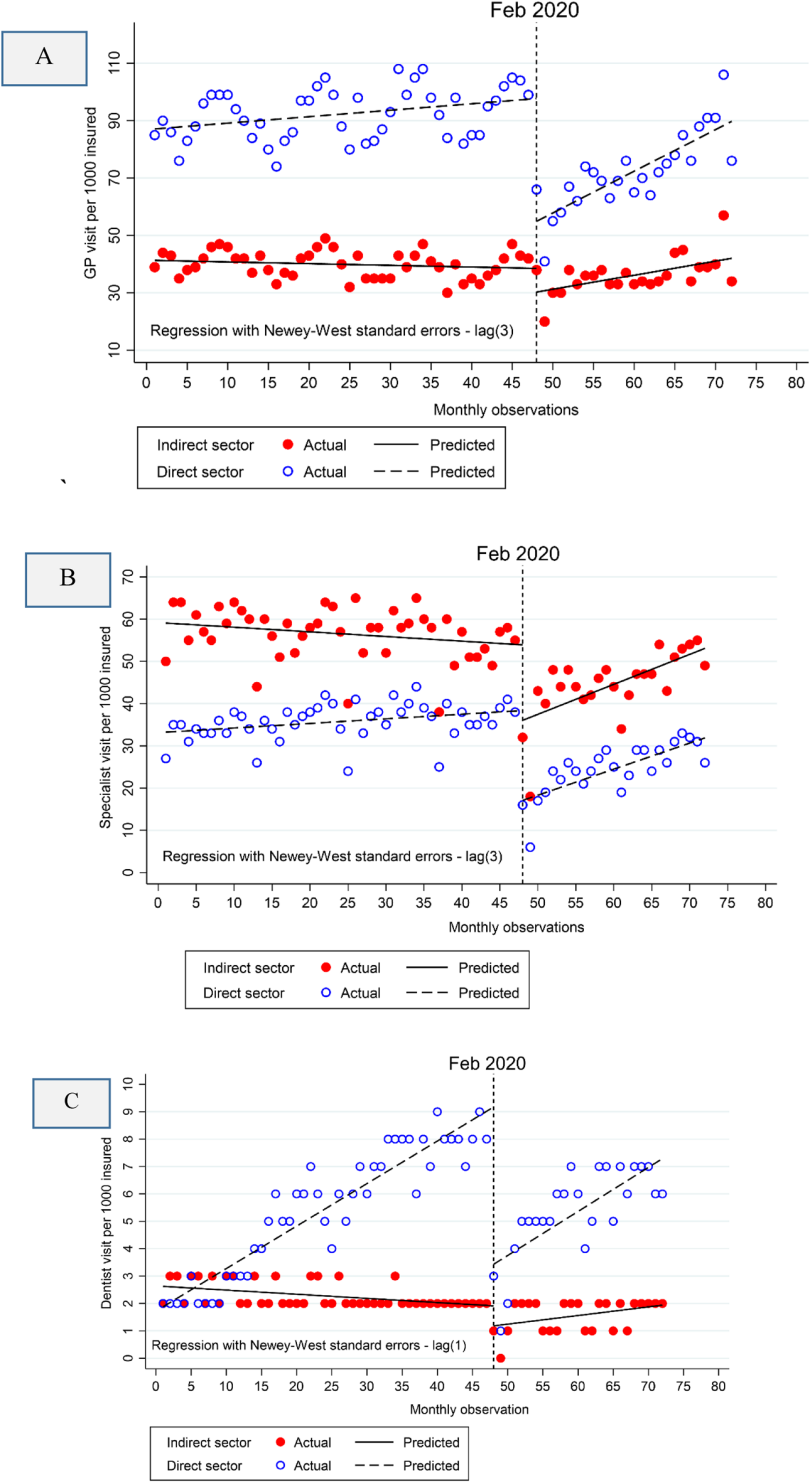
Multiple group ITSA with Newey–West standard errors and three lags for visits of GPs (**A**), specialists (**B**) and dentists (**C**) per 1000 insured. Note: The COVID-19 pandemic outbreak occurred in February 2020.

The results of the multiple group ITSA for the outcome variables (GP visit, specialist visit, and dentist visit) are reported in Table 3. The results reveal a significant decrease of 42.76 [95% CI −51.15 to −34.37] GP visits per 1000 insured people in the SSO's direct sector upon the onset of the pandemic, followed by decreases of 21.28 [95% CI −26.42 to −16.15] for specialists and 5.74 [95% CI −0.15 to −4.32] for dentists. Before the pandemic, the number of GP visits per 1000 insured was 45.79 [95% CI −52.69 to −38.89] higher in the direct sector compared to the indirect sector. However, in this same period, the number of specialist visits and dentist visits per 1000 insured was lower in the direct sector than the indirect sector, by 25.84 [95% CI 22.87 to 28.82] and 0.75 [95% CI 0.12 to 1.36] visits, respectively. In the months following the pandemic and for the direct sector, there was a significant increase of 1.22 [95% CI 0.75 to 1.71], 0.51 [95% CI 0.23 to 0.79], and 0.005 [95% CI −0.08 to 0.09] for GP, specialists, and dentists, respectively. When considering the indirect sector, the differences in pre-trend values between the direct and indirect sectors for GPs, specialists, and dentists were −0.28 [95% CI −0.54 to −0.02], −0.22 [95% CI −0.33 to −0.10], and −0.17 [95% CI −0.19 to −0.14], respectively. The changes in these differences between pre-pandemic and the pandemic periods were −0.67 [95% CI −1.22 to −0.12] for GP visits, 0.31 [95% CI −0.17 to 0.79] for specialists, and 0.04 [95% CI −0.05 to 0.13] for dentists. Among these changes, only the difference for GP visits was statistically significant (p-value < 0.05). Visual representations of these results can be found in Fig. 2A–C.

**Table 3:** Multiple group analysis of the effect of the COVID-19 pandemic on visits to GP, specialists, and dentists among those insured by the Social Security Organization in the direct sector versus indirect sector.

Sectors			GP^a^ visit		Specialist visit		Dentist visit	
			Coefficient (95% CI^b^)	p-value	Coefficient (95% CI)	p-value	Coefficient (95% CI)	p-value
Direct sector	Before the pandemic	Mean value at the baseline (β_0_)	87.13 (80.95 to 93.31)	< 0.001	33.24 (31.56 to 34.91)	< 0.001	1.87 (1.28 to 2.47)	< 0.001
		Pre-trend (β_1_)	0.22 −0.00 to 0.45)	0.056	0.11 (0.04 to 0.18)	0.002	0.15 (0.13 to 0.18)	< 0.001
	during the pandemic	During-level change (β_2_)	−42.76 (−51.15 to −34.37)	< 0.001	−21.28 (−26.42 to −16.15)	< 0.001	−5.7 (−7.15 to −4.32)	< 0.001
		During-trend change (β_3_)	1.22 (0.75 to 1.71)	< 0.001	0.51 (0.23 to 0.79)	< 0.001	0.005 (−0.08 to 0.09)	0.893
Indirect sector relative to direct sector	Before the pandemic	Pre-level difference (β_4_)	−45.79 (−52.69 to −38.89)	< 0.001	25.84 (22.87 to 28.82)	< 0.001	0.75 (0.12 to 1.36)	0.006
		Pre-trend difference (β_5_)	−0.28 (−0.54 to −0.02)	0.036	−0.22 (−0.33 to −0.10)	< 0.001	−0.17 (−0.19 to −0.14)	< 0.001
	During the pandemic	During-level difference (β_6_)	34.44 (24.81 to 44.08)	< 0.001	3.41 (−5.87 to 12.69)	0.469	5.01 (3.48 to 6.53)	< 0.001
		Change in slope difference pre-to during (β_7_)	−0.67 (−1.22 to −0.12)	0.016	0.31 (−0.17 to 0.79)	0.206	0.04 (−0.05 to 0.13)	0.364
Model significance		F	171.02		160.3		154.3	
		P-value	< 0.001		< 0.001		< 0.001	

a: GP is the general physician; CI: confidence interval. ${\beta }_{0}$ estimates intercept (starting level of Y in direct sector); ${\beta }_{1}$ estimates pre-pandemic slope (trend in Y over time in direct sector); ${\beta }_{2}$ estimates the level change in Y in direct sector in the first month of the pandemic; ${\beta }_{3}$ is the difference in slope changes between during the pandemic and pre-pandemic periods (change in trend in Y during the pandemic in direct sector); ${\beta }_{4}$ estimates the difference in baseline Y between direct and indirect sectors; ${\beta }_{5}$ estimates the difference in pre-pandemic trends between direct and indirect sectors; ${\beta }_{6}$ estimates the difference in level change during the pandemic between direct and indirect sectors and ${\beta }_{7}$ estimates the differences in slope changes from pre-pandemic to pandemic periods between direct and indirect sectors.

## Discussion

Because of measures to reduce the spread of coronavirus this had an impact on the utilization of routine health care. It is vital for policy makers to realize the extent and severity of how this impacted on health service usage across a range of care pathways^35,36^. This is even more important for health system in a lower- and middle-income countries (LMICs) such as Iran where the health system is less resilient against crisis. We utilized an ITSA method to evaluate the impact of the pandemic on physician and dentist visit rates. The results of this study demonstrate the negative impact of the pandemic on receiving routine visits among a representative proportion of Iran’s population.. Similar to previous studies^3,37–39^.

Compared to pre-pandemic levels, the descriptive results show that monthly GP, specialist, and dentist visits per 1000 insured decreased by 18%, 24%, and 14.1% respectively in the whole sector (including both direct and indirect sectors) during the pandemic period. During a pandemic, there are several reasons why people may visit doctors, specialists, and dentists less frequently. One reason why usage may decline is because of government's policies such as stay at home orders and physical distancing. . These measures aim to reduce the spread of the virus by minimizing contact between people. Another reason is the fear and anxiety of contracting the disease. Many individuals may be hesitant to seek medical care, especially for non-emergency issues.. This fear can lead to a decrease in healthcare utilization. Additionally, the healthcare system may be strained by the increased demand for COVID-19-related care limiting resources for other types of health care. This strain can affect the availability and accessibility of non-emergency healthcare services, resulting in fewer visits^40,41^.

Consistent with our findings that the pandemic led to decreased health service utilization, with the significant declines in the first month, studies from other countries and within Iran have also shown that the COVID-19 pandemic resulted in reduced use of health services across multiple sectors. A study on insured patients in the US found that during the COVID-19 outbreak^42^, while dental care utilization among privately insured patients recovered to pre-pandemic levels by August 2020, use among publicly insured patients was still 7.54% lower than before the pandemic. .In another study in Iran, it was found that in the short-term, utilization of private laboratory, radiology, medication, and hospital admissions decreased significantly by 18,066, 8210, 135,445, and 1086 times, respectively^37^. Mahmood pour- Azari et al.^3^ in their study in west of Iran found significant initial decreases in hospitalizations (38.1 per 10,000), emergency department visits (191.6 per 10,000), and outpatient visits (168.6 per 10,000) during the first COVID-19 outbreak month. While dental care utilization among privately insured patients recovered back to pre-pandemic levels by August 2020 after significant declines earlier in the pandemic, dental care use among publicly insured patients remained 7.54% below pre-COVID-19 levels at the same time point in August 2020^43^.

An interesting finding of the study is that, the multiple group analysis further showed differential effects between the direct and indirect sectors. GP and specialist visits in the direct sector experienced greater initial declines but also larger subsequent monthly increases compared to the indirect sector. However, for dentist visits, the immediate reduction was smaller in the direct sector while the indirect sector had a larger rebound increase initially before leveling off. This observation suggests a potential scenario where the medical centers under the SSO have increased utilization levels more quickly than other health care providers .. An additional plausible explanation for this discrepancy could be attributed to the scale of service provision. The SSO's owned medical centers, numbering 75 with 11,593 beds, are relatively smaller compared to the 664 affiliated hospitals of the Ministry of Health and Medical Education (MOHME), boasting a total of 105,676 beds. Consequently, at the national scale, there are likely more influential factors at play to mitigate the adverse effects of a pandemic.

The study findings indicate that the Iranian healthcare system lacks adequate resilience against crises such as the COVID-19 pandemic, as evidenced by the significant reductions in routine health services utilization like physician and dentist visits during the pandemic and the failure to return to pre-pandemic levels even after 25 months; suggesting that there may be unmet needs that will manifest themselves in the long run.

One key recommendation to improve the resilience of the healthcare system against future crises for Iranian policymakers is to explore alternative service delivery models, such as virtual and remote services. This approach would establish an agile system that minimizes delays in providing essential services. Looking at international examples from other countries (although mostly from high-income-countries), that have successfully compensated for the gap in in-person services by embracing virtual care. For instance, in Canada, the use of virtual care accounted for 50% or more of ambulatory care visits over the first 9 months of the COVID-19 pandemic^44^, with a similar trend seen in the U.S.^45^, where telemedicine services increased 154% in March 2020 compared to March 2019. These cases provide valuable lessons for LIMCs countries including Iran to enhance their telemedicine and virtual capabilities, meeting unmet health service demands during the pandemic and beyond.

It's important to consider that the delay in returning to pre-pandemic levels of visits might be explained by patients seeking healthcare through alternative channels, beyond the scope of both direct and indirect SSO services. This shift in healthcare-seeking behavior could contribute to the observed delay in returning to pre-pandemic utilization patterns within the SSO system.

This study has many strengths including having access to data over time and employing a robust estimation approach. In this study, 47 time points were considered part of the pre-pandemic baseline and 25 time points were considered part of the pandemic period. Other studies have typically used fewer time points, which has been noted as a limitation. By including many time points before and after the pandemic emerged, our study provides a more comprehensive picture of how visit patterns changed during the pandemic period. However, the study findings might be limited to the people who are under the SSO’s cover., It is worth noting that the SSO serves approximately 54% of the country’s population. Second, the data used in this study was aggregated and not at individual-level data, it is expected that individual’s demographical and health profiles, may affect healthcare utilization. These factors should be examined in future studies to understand how they influence the utilization patterns observed in this analysis. On the other hand, our study was conducted using robust and well-established methods that involved multiple time points and a large study population..

## Conclusion

This interrupted time series analysis indicates that the COVID-19 pandemic led to substantial reductions in healthcare utilization, including decreased physician and dentist visits, among those insured by the SSO in Iran. Immediately after the pandemic, GP, specialist, and dentist visits per 1000 insured dropped significantly. While visit rates partially recovered over time, they remained below pre-pandemic levels throughout the 25-month during the pandemic period analyzed. The nation's healthcare system appears to have struggled in effectively addressing the challenges posed by the pandemic, particularly in meeting the demand for physician and dentist visits. One potential explanation for this deficiency is the absence of a comprehensive and well-established nationwide infrastructure for telemedicine and virtual consultations. There is a pressing need for the country's health system to advance and establish a widespread network for virtual healthcare services. Additionally, establishing a prioritization agenda is crucial to effectively manage crisis such as COVID-19 pandemic while ensuring the continued provision of routine services, including routine visits.

### Supplementary Information

Supplementary Information.

## Data Availability

The datasets generated and analyzed during the current study are available from the corresponding author upon reasonable request.
