# Supplementary material for: Time series analysis of COVID-19's impact on physician and dentist visits in Iran
Source: Sci Rep. 2024 Jul 16;14:16453. doi: 10.1038/s41598-024-67238-9 (PMC11252254; doi:10.1038/s41598-024-67238-9)
Supplement: Supplementary file 1 — Supplementary Information. [file 41598_2024_67238_MOESM1_ESM.docx]

**Healthcare system and health insurance in Iran**

- The structure and provision of health care: Three main sectors - public, private, and non-profit/charity provide the healthcare services in Iran. The public sector plays a big role in providing- primary, secondary, and tertiary care. The private sector mainly provides secondary and tertiary care in urban areas. Non-government organizations (NGOs) alongside charities also provide health services for patients with chronic illnesses (e.g. diabetes )or severe diseases (e.g. cancer) (1). At national level the Ministry of Health and Medical Education (MOHME) is governor of health system. At regional (provincial) level the medical universities which are affiliated to the MOHME oversees healthcare. There is at least one medical university in each of Iran's 32 provinces. They supervise public healthcare and monitor private providers. In addition to affiliated medical centres to MOHME there are some other public entities which have their own medical centres such as the Ministry of Petroleum, the Ministry of Defense, and the SSO. But they still must follow MOHME regulations.
- Healthcare financing: funding comes from the government budget (nearly 20% of total health spending), public health insurance (approximately 30% of total health spending), private health insurance (less than 10 % of total health spending), and out-of-pocket payments by individuals (nearly 40% of total health spending) (1, 2, 3, 4, 5, 6). There are four public insurers:

1. Iranian Health Insurance Organization (IHIO) - covers civil servants, rural residents, a proportion of self-employed people, students, and remained uninsured people under the universal health insurance scheme
2. SSO- covers formal sector employees and a big proportion of self-employed workers: The SSO provides health insurance coverage to over 44 million people in Iran (exactly 44,150,091 in 2021), which represents more than 50% of the total population.
3. Armed Forces Medical Service Organization - covers military members
4. Imam Khomeini Relief Foundation - covers poor and deprived population.

Some other organizations like oil companies and banks have their own insurance too. There is also some private health insurance.

With regard of SSO, the SSO in Iran was created in 1975 to provide health insurance coverage and healthcare services to employed people. It is the second largest healthcare provider in Iran after the MOHME. The total number of SSO’s own hospitals in 2021 was around 78, the total number of hospital beds was nearly 9000, the number of clinics (general, private, specialized, polyclinic, etc.) was 312, and there were 5 surgical centers or Day Clinics. The SSO provides health services to insured patients in two main ways- direct and indirect sectors. In the direct sector, healthcare facilities owned by SSO including hospitals, clinics, pharmacies, rehabilitation centers, etc. provide free treatment and medicines to patients. In the indirect sector, the SSO has contracts with hospitals/medical centrs affiliated with medical universities, private hospitals, labs, physicians' offices, etc. to provide care to insured patients. Patients often prefer the direct sector because it is free. But this causes long wait times. Patients who can afford it may go to the indirect sector for quicker access. The SSO is financed through insurance contributions from insured members and employers. The government also provides subsidies to the organization. (4, 5, 7).

1. Mehrdad R. Health system in Iran. JMAJ. 2009;52(1):69-73.

2. Hajizadeh M, Nghiem HS. Hospital care in Iran: an examination of national health system performance. International Journal of Healthcare Management. 2013;6(3):201-10.

3. Hajizadeh M, Connelly LB. Equity of health care financing in Iran: the effect of extending health insurance to the uninsured. Oxford Development Studies. 2010;38(4):461-76.

4. <https://landinfo.no/wp-content/uploads/2020/08/Report-Iran-Welfare-system-12082020.pdf>.

5. Doshmangir L, Bazyar M, Rashidian A, Gordeev VS. Iran health insurance system in transition: equity concerns and steps to achieve universal health coverage. International Journal for Equity in Health. 2021;20(1):37.

6. Dehnavieh R, Rahimi H. Basic health insurance package in Iran: revision challenges. Iranian journal of public health. 2017;46(5):719.

7. Davari M, Haycox A, Walley T. The Iranian health insurance system; past experiences, present challenges and future strategies. Iranian journal of public health. 2012;41(9):1.
